# Evolution of the Mesenteric Mass in Small Intestinal Neuroendocrine Tumours

**DOI:** 10.3390/cancers13030443

**Published:** 2021-01-25

**Authors:** Anela Blažević, Tessa Brabander, Wouter T. Zandee, Johannes Hofland, Gaston J. H. Franssen, Marie-Louise F. van Velthuysen, Richard A. Feelders, Wouter W. De Herder

**Affiliations:** 1 Department of Internal Medicine, Sector Endocrinology, ENETS Center of Excellence, Erasmus University Medical Center and Erasmus MC Cancer Institute, 3015 GD Rotterdam, The Netherlands; w.t.zandee@umcg.nl (W.T.Z.); j.hofland@erasmusmc.nl (J.H.); r.feelders@erasmusmc.nl (R.A.F.); w.w.deherder@erasmusmc.nl (W.W.D.H.); 2 Department of Radiology & Nuclear Medicine, ENETS Center of Excellence, Erasmus University Medical Center and Erasmus MC Cancer Institute, 3015 GD Rotterdam, The Netherlands; t.brabander@erasmusmc.nl; 3 Department of Endocrinology, University Medical Center Groningen and University of Groningen, 9700 RB Groningen, The Netherlands; 4 Department of Surgery, ENETS Center of Excellence, Erasmus University Medical Center and Erasmus MC Cancer Institute, 3015 GD Rotterdam, The Netherlands; g.franssen@erasmusmc.nl; 5 Department of Pathology, ENETS Center of Excellence, Erasmus University Medical Center and Erasmus MC Cancer Institute, 3015 GD Rotterdam, The Netherlands; m.vanvelthuysen@erasmusmc.nl

**Keywords:** neuroendocrine tumour, mesenteric metastases, progression, radiology, CT scan, PRRT, RECIST 1.1

## Abstract

**Simple Summary:**

Around two-thirds of patients with small intestinal neuroendocrine tumours are present with a metastatic mesenteric mass. This mass is known to cause intestinal complications, however, little is known on its development over time in the era of targeted therapy. Therefore, we conducted a retrospective study to assess the growth and response to therapy. We found that the growth of the mesenteric mass was detectable in 13.5% over a median time of 3.4 years and peptide receptor radionuclide therapy resulted in size reduction in only 3.8%. This site-specific static growth behavior is important to note when assessing disease progression and therapeutic options.

**Abstract:**

Background: A metastatic mesenteric mass is a hallmark of small intestinal neuroendocrine tumours (SI-NETs). However, little is known on its development over time. Therefore, we conducted a study to assess the evolution of a SI-NET-associated mesenteric mass over time. Methods: Retrospectively, 530 patients with proven SI-NET were included. The presence and growth of a mesenteric mass was assessed using RECIST 1.1 criteria on every consecutive CT-scan until the end of follow-up or resection. Results: At baseline, a mesenteric mass was present in 64% of the patients, of whom 13.5% showed growth of the mesenteric mass with a median time to growth of 40 months. Male gender was the only independent predictor of growth (OR 2.67). Of the patients without a mesenteric mass at the first evaluation, 2.6% developed a pathological mesenteric mass. Treatment with peptide receptor radionuclide therapy (PRRT; *N* = 132) resulted in an objective size reduction of the mesenteric mass in 3.8%. Conclusion: The metastatic mesenteric mass in SI-NETs has a static behavior over time. Therefore, site-specific growth behavior should be taken into account when selecting target lesions and assessing disease progression and therapeutic response. PRRT appears not to be effective for size reduction of the mesenteric mass.

## 1. Introduction

**Figure 1:**
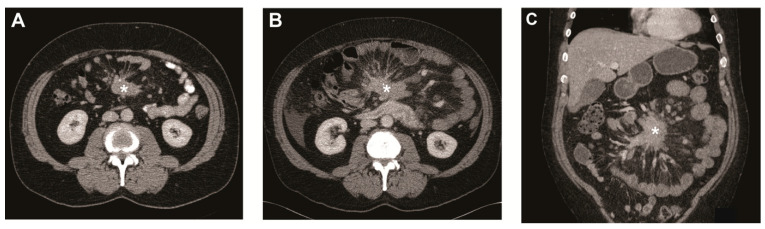
Metastatic mesenteric mass and surrounding fibrosis over time. (**A**) Transverse image of CT scan at baseline showing mesenteric mass (asterisk) with radiating strands of fibrotic tissue. Transverse (**B**) and coronal image (**C**) of CT scan after 5 years showing the mesenteric mass (asterisk) growth of >20% on the short axis.

Small intestinal neuroendocrine tumours (SI-NETs) are often diagnosed at an advanced stage with the mesentery being one of the dominant metastatic sites [1,2,3]. The metastatic mass is known to induce fibrosis in the surrounding mesentery (Figure 1), which can cause serious complications such as bowel obstruction and ischemia [1,2,3,4,5]. Even though the survival of patients with advanced SI-NETs has improved due to targeted treatment options such as somatostatin analogues (SSAs), everolimus, and peptide receptor radionuclide therapy with ^177^Lu-DOTATATE (PRRT), treatment options for intestinal complications due to mesenteric metastasis and fibrosis remain limited to primarily intestinal resection or bypass [1,5,6,7]. As a preventive treatment, the current European Neuroendocrine Tumor Society (ENETS) guideline advises to consider prophylactic palliative surgery in SI-NET patients with mesenteric metastasis [8]. However, not all patients develop abdominal complications, approximately 30% of patients with mesenteric disease are asymptomatic. Moreover, there is increasing evidence that prophylactic palliative resection of the primary tumour and mesenteric mass does not result in an overall improved outcome [2,9,10]. Currently, there is no method to identify patients with a high risk of progressive mesenteric disease that may benefit from prophylactic palliative surgery. Increased knowledge on the clinical course of the SI-NET-associated mesenteric mass is essential in order to develop these criteria. Furthermore, understanding of the clinical course and factors associated with progressive disease could point to underlying pathways and aid the development of novel therapeutic options.

Therefore, the aim of this study was to obtain more insight in the clinical course of metastatic mesenteric masses in SI-NETs. To this end, we have used routinely obtained CT scans, and assessed the growth of the mesenteric mass over time and tried to identify patients at high risk for disease progression based on clinical criteria.

## 2. Results

### 2.1. Patient Characteristics

From a cohort of 635 patients with SI-NETs, 530 patients had at least two accessible CT scans and were included for analysis. Of the excluded 105 patients with less than two accessible CT scans, 70 were once assessed and further follow-up was performed in another center, often outside the Netherlands, and 35 had no analyzable CT scans due to other reasons. Baseline characteristics are shown in Table 1. A mesenteric mass was present in 64.2% of patients at baseline. The patients with mesenteric metastases were older, had a more advanced disease as expressed by the disease stage, presence of liver metastases, and tumour marker levels. Additionally, there was a male predominance (*p* ≤ 0.001).

### 2.2. Mesenteric Metastases Over Time

The evolution of the mesenteric metastases is shown in Table 2. In the overall group, 9.2% of patients showed the development or growth of the mesenteric mass. The median follow-up time was 34 months (range 1–186; interquartile range (IQR) 14–61). There was no significant difference in the follow-up time between patients with and without a mesenteric mass, and patients with and without growth. Patients with a mesenteric mass at baseline (*N* = 340), showed growth in 13.5% (*N* = 46) with a median time to growth of 40 months (range 4–134; IQR 15–61). In contrast, patients without a mesenteric mass at baseline (*N* = 190) rarely developed an objective mesenteric disease (*N* = 5, 2.6%) with an approximately equal time to development (range 7–113).

To obviate the bias induced by inclusion of patients at referral to a tertiary center after the initial surgical treatment, we performed a subgroup analysis of patients with a follow-up from before the first abdominal surgery and found no significant difference in the growth rate or time to growth (see Supplementary Materials).

### 2.3. Predictors of Growth

To find predictors of mesenteric mass growth, we analyzed patients with a mesenteric mass at baseline. Patients that underwent resection of the mesenteric mass (*N* = 11) had a significant shorter follow-up time compared to the overall follow-up time (median follow-up time 7 vs. 34 months, respectively, *p* = 0.01). As this follow-up was also notably shorter than the median time to growth of mesenteric masses (7 vs. 40 months, respectively), we excluded these patients from this analysis. To find predictors of growth, we performed the univariate analysis of the baseline patients and disease characteristics and the size of the mesenteric mass. We found male gender and tumour grade to be predictors of growth (Table 3). Other baseline characteristics such as age or tumour markers were not significantly associated with growth. When we combined the significant predictors in a multivariate model, only male gender remained an independent predictor of mesenteric mass growth.

### 2.4. Received Treatments and Mesenteric Mass Growth

In our cohort, patients received SSAs in 82.8% and PRRT in 26.4% of cases as shown in Table 1. Patients with a mesenteric mass received more often SSAs, even when corrected for the ENETS disease stage (OR 3.87, 95% CI: 2.25–6.63, *p* < 0.001). There was no significant difference in the percentage of patients that received PRRT. Next, we assessed the difference in the treatment received by patients with and without growth of the mesenteric mass. There was no difference regarding the rate of SSAs use (both 91%, *p* = 1.000) or PRRT administration (40% vs. 39%, *p* = 0.871, respectively).

We have also assessed surgical treatments. As shown in Table 1, patients with a mesenteric mass less often received surgery. However, palliative surgery for symptomatic control is performed in approximately the same percentage of patients (26.6% in patients with a mass vs. 29.1% in patients without a mass, *p* = 0.77). As the study had a long time-frame, we also assessed if the disease management changed over the years. We divided the cohort in four groups based on data of diagnosis (<2008, 2008–2012, 2012–2016, and >2016) and found no significant shift in the percentages of patients operated or in the indications for surgery. Finally, there was also an equal percentage of patients with and without growth that underwent palliative surgery for symptomatic control (33% vs. 26%, respectively, *p* = 0.458).

Of the 132 patients with a mesenteric mass that received PRRT, an objective response (≥30% reduction of the sum of diameters of all target lesions) was noted in 12.9%. In contrast, a ≥30% reduction of the mesenteric mass was only observed in 3.8% of the patients. The five patients with an objective mesenteric mass reduction (range 32–50% of the diameter on the short axis) showed no growth of the mass before PRRT and the timing between diagnosis and PRRT ranged from 2 to 96 months.

## 3. Discussion

In the current study, we analyzed the evolution of mesenteric metastases in a large cohort of patients with SI-NETs with a median follow-up time of 34 months. In our cohort, a metastatic mesenteric mass was present in 64% of the SI-NET patients. During follow-up, growth of the mesenteric mass was noted in a minority (13.5%) and when present, the time to growth was remarkably long with a median of 40 months (see Figure 1). Moreover, the development of a mesenteric mass in patients without mesenteric disease at baseline was very rare and only observed in five patients (2.6%).

In order to gain more insight in the mechanisms underlying mesenteric disease progression in SI-NETs, we assessed patient and disease characteristics as potential predictors of growth. In the multivariate analysis, only male gender remained a significant predictor of growth. This finding suggests an effect of sex on SI-NETs and mesenteric metastasis, possibly mediated by steroid hormone receptors [11,12,13]. However, further research is necessary to understand the relevance of this finding.

When analyzing the treatment response, the static growth pattern of mesenteric metastases could also be observed. When we assessed patients with a mesenteric mass that received PRRT, we found an objective response in 12.9%. This is comparable with results from the NETTER-1 trial (CR+PR: 18%) [6]. However, when we exclusively assessed the effect on the mesenteric mass, we found that only 3.8% of patients had an objective response. Therefore, PRRT does not seem to be an effective treatment to reduce the SI-NET-associated mesenteric mass size. However, PRRT might still have an effect on the surrounding fibrosis and clinical symptoms [14,15].

These outcomes illustrate the limitations of solely relying on RECIST 1.1 criteria to assess the disease progression and therapeutic effect in SI-NETs. Due to the highly static behavior of the mesenteric mass, patients with a dominant mesenteric disease might be falsely classified as a stable disease and therefore not receive the proper treatment for the progressive disease. Moreover, these patients might be falsely classified as non-responsive to treatments such as PRRT. Therefore, we believe that when assessing the disease development in SI-NETs, site-specific growth behavior should be taken into account and the SI-NET-associated mesenteric mass should preferably not be included as target lesion for determining the disease progression and treatment response.

Our study has some limitations to note, including that it is performed in a single, tertiary referral center. As a result, patients often received a first medical or surgical treatment before referral. However, a subgroup analysis of patients with follow-up from before the first surgical intervention did not show a difference in the growth rate. Furthermore, most patients received targeted medical treatments, such as SSAs, that could have inhibitory effects and alter the growth behavior of the mesenteric mass. However, as this reflects the current management strategy, we believe our results accurately reflect the growth behavior of mesenteric masses in the era of targeted treatments.

## 4. Methods

### 4.1. Patients

Patients from the NET-database, which encompassed all NET patients treated between 1993 and 2016 in the Erasmus Medical Center in Rotterdam, were included if they had proven SI-NET and ≥2 contrast-enhanced CT scans were available. As the study was retrospectively performed with anonymized data, according to the Central Committee on Research involving Human Subjects (CCMO), no approval from the Ethics Committee in the Netherlands was required. The disease characteristics and tumour markers were determined at the time of diagnosis or, if not available, the first measurement at our center was used. An extensive description of the methods used for tumour marker measurement was published previously [16]. To assess the development over time, we divided the cohort based on the date of diagnosis. The cut-offs were based on the publication data of the sequential ENETS guidelines resulting in four groups: <2008 (*N* = 188), 2008–2012 (*N* = 161), 2012–2016 (*N* = 150), >2016 (*N* = 31) [8,17,18].

### 4.2. Imaging

Radiological features were assessed by means of contrast-enhanced CT. A mesenteric node of ≥ 10 mm on the short axis was considered a metastatic mass. Growth of the largest mesenteric mass was assessed on all the available CT scans in accordance with RECIST 1.1 criteria until the end of follow-up, significant growth of mesenteric mass, or resection of mesenteric mass. Significant growth was determined if at least a 20% increase of the diameter of the short axis of the mesenteric mass was measured. In addition, the absolute increase needed to be at least 5 mm [19]. The effect of PRRT was evaluated until 12 months after the last cycle, also in accordance with RECIST 1.1 criteria. Both patients with a complete response (CR; disappearance of all target and non/target lesions) and partial response (PR; at least a 30% decrease in the sum of diameters of targets lesion) were included in the objective response category [19]. Therefore, when assessing only the mesenteric mass, patients were considered to have an objective response if there was a disappearance of the mesenteric mass or decrease of at least 30% of the diameter on the short axis.

### 4.3. Statistics

The SPSS software (version 21 for Windows, SPSS Inc.) was used to perform the analyses. Data were presented as median, range, and IQR (25th–75th percentiles) or a percentage with count. Continuous data were compared using the unpaired *t*-test, Mann-Whitney U test, or ANOVA as appropriate. For post-hoc multiple comparison, the Dunnett’s T3 test was used as equal variances were not assumed. The Fisher exact test was performed for comparison of categorical data. Odds ratios (OR) with a 95% confidence interval (CI) were determined using the univariate and multivariate logistic regression. A *p*-value of <0.05 was considered statistically significant.

## 5. Conclusions

In this study, the data have important clinical implications as they demonstrate the static behavior of the SI-NET-associated mesenteric mass, which should be taken into account when selecting target lesions and assessing disease progression, therapeutic response, and treatment options. PRRT appears not to be effective for size reduction of the mesenteric mass.

## Figures and Tables

**Table 1:** Characteristics.

	All Patients (*N* = 530)	Patients with Mesenteric Mass ≥10 mm (*N* = 340)	Patients Without Mesenteric Mass ≥10 mm (*N* = 190)	*p*-Value
**Patient Characteristics**				
Age	60.3 (52.1–68.3)	61.6 (54.1–69.7)	57.1 (49.8–65.5)	<0.001
Male	53.2% (*N* = 282)	58.8% (*N* = 200)	43.2% (*N* = 82)	0.001
**Disease Characteristics**				
Tumour grade				0.105
Grade 1	50.0% (*N* = 265)	48.8% (*N* = 166)	52.1% (*N* = 99)	
Grade 2	26.4% (*N* = 140)	29.7% (*N* = 101)	20.5% (*N* = 39)	
Grade 3	2.1% (*N* = 11)	1.8% (*N* = 6)	2.6% (*N* = 5)	
Missing	21.5% (*N* = 114)	19.7% (*N* = 67)	24.7% (*N* = 47)	
ENETS disease stage				<0.001
Stage I / II	2.8% (*N* = 15)	0.6% (*N* = 2)	6.9% (*N* = 13)	
Stage III	20.9% (*N* = 111)	17.6% (*N* = 60)	26.8% (*N* = 51)	
Stage IV	75.8% (*N* = 402)	81.2% (*N* = 276)	66.3% (*N* = 126)	
Liver metastasis	71.1% (*N* = 377)	77.1% (*N* = 262)	60.5% (*N* = 115)	<0.001
CgA (μg/L)	205.0 (90.5–748.5)	244.5 (109.5–826.0)	136.5 (63.0–546.3)	<0.001
5-HIAA (µmol/24 h)	107.9 (42.4–439.2)	154.2 (63.4–519.0)	51.6 (24.8–241.4)	<0.001
**Treatments**				
SSAs	82.8% (*N* = 439)	91.2% (*N* = 310)	67.9% (*N* = 129)	<0.001
PRRT	44% (*N* = 233)	46.8% (*N* = 159)	38.9% (*N* = 74)	0.08
Surgery	70.6% (*N* = 374)	63.2% (*N* = 215)	83.7% (*N* = 159)	<0.001
Curative	23.9% (*N* = 122)	14.2% (*N* = 48)	43.0% (*N* = 74)	
Palliative for symptom control	27.5% (*N* = 140)	26.6% (*N* = 90)	29.1% (*N* = 50)	
Prophylactic palliative	18.8% (*N* = 96)	18.9% (*N* = 64)	18.5% (*N* = 32)	
Indication not reported	3.1% (*N* = 16)	2.5% (*N* = 13)	1.7% (*N* = 1.7%)	

Numerical data are median with an interquartile range in brackets. Categorical data are percentages with a count in brackets. CgA: Serum chromogranin A, normal range <94 μg/L, 5-HIAA: Urinary 5-HIAA excretion, normal range <50 μmol /24 h.

**Table 2:** Evolution of mesenteric mass over time.

	All Patients (*N* = 530)	Patients with Mesenteric Mass ≥10 mm (*N* = 340)	Patients without Mesenteric Mass ≥10 mm (*N* = 190)	*p*-Value
No growth	88.3% (*N* = 468)	83.2% (*N* = 283)	97.4% (*N* = 185)	<0.001
Growth *	9.2% (*N* = 51)	13.5% (*N* = 46)	2.6% (*N* = 5)	
Resection	2.1% (*N* = 11)	3.2% (*N* = 11)	N/A	

* Growth assessed by RECIST 1.1 criteria and compared to the baseline CT scan. In the case of mesenteric mass at baseline, growth is defined as an increase of ≥20% and ≥5 mm on the short axis of the dominant mesenteric mass. In the case of no mesenteric mass at baseline, growth is defined as the development of a mesenteric node of ≥10 mm on the short axis.

**Table 3:** Predictors of growth in patients with mesenteric mass (*N* = 329).

	Univariate			Multivariate		
	OR	95% CI	*p*-Value	OR	95% CI	*p*-Value
Age	0.98	0.95–1.01	0.107	NS		
Male	2.15	1.06–4.32	0.033	2.67	1.19–5.99	0.017
Tumour grade						
Grade 1	Reference			Reference		
Grade 2	0.43	0.19–0.99	0.048	0.43	0.19–1.01	0.051
Grade 3	0.97	0.11–8.64	0.978	1.24	0.13–11.53	0.853
ENETS disease stage						
Stage I and II	Reference					
Stage III and IV	0.16	0.01–2.54	0.192	NS		
CgA (μg/L)	1.00	1.00–1.00	0.791	NS		
5-HIAA (µmol/24 h)	1.00	1.00–1.01	0.877	NS		
Liver metastasis	0.85	0.41–1.78	0.673	NS		
Mesenteric mass size (mm)	0.99	00.96–1.02	0.438	NS		

OR: Odds ratio; 95% CI, 95% confidence interval; NS: Non-significant in univariate analysis; CgA: Serum chromogranin A normal range <94 μg/L; 5-HIAA, urinary 5-HIAA excretion, normal range <50 μmol/24 h.

## Data Availability

The data that support the findings of this study are available from the corresponding author upon reasonable request.

## Supplementary Materials

The following are available online at https://www.mdpi.com/2072-6694/13/3/443/s1, Table S1: Comparison of evolution of mesenteric mass over time in all patients, Table S2: Comparison of evolution of mesenteric mass over time in patients with mesenteric mass ≥ 10 mm at baseline, Table S3: Comparison of evolution of mesenteric mass over time in patients without mesenteric mass ≥ 10 mm at baseline.
