# Supplementary material for: Evolution of the Mesenteric Mass in Small Intestinal Neuroendocrine Tumours
Source: Cancers (Basel). 2021 Jan 25;13(3):443. doi: 10.3390/cancers13030443 (PMC7865677; doi:10.3390/cancers13030443)
Supplement: Supplementary file 1 [file cancers-13-00443-s001.pdf]

Supplemental data

Subgroup analysis of patients with follow-up before first abdominal surgery including patients who did not receive abdominal surgery during follow-up (N = 282) versus complete cohort (N = 530). The subgroup analysis has a median follow-up time of 32.3 months (IQR 12.0– 62.1) with a median time to growth of 38.5 months (IQR 12.3 – 73.2).

Supplement table 1. Comparison of evolution of mesenteric mass over time in all patients.

	Complete cohort (N = 530)	Subgroup (N = 282)	*P-value*
No growth	88.3% (N = 468)	86,9% (N = 245)	0.453
Growth*	9.2% (N = 51)	9,6% (N = 27)	
Resection	2.1% (N = 11)	3,5% (N = 10)	

Supplement table 2. Comparison of evolution of mesenteric mass over time in patients with mesenteric mass ≥ 10 mm at baseline.

	Complete cohort (N = 340)	Subgroup (N = 234)	*P-value*
No growth	83.2% (N = 283)	85.5% (N = 200)	0.426
Growth*	13.5% (N = 46)	10,3% (N = 24)	
Resection	3.2% (N = 11)	4.2% (N = 10)	

Supplement table 3. Comparison of evolution of mesenteric mass over time in patients without mesenteric mass ≥ 10 mm at baseline.

	Complete cohort (N = 190)	Subgroup (N = 48)	*P-value*
No growth	97.4% (N = 185)	93.9% (N = 45)	0.214
Growth*	2.6% (N = 5)	6,1% (N = 3)	

*Growth assessed by RECIST 1.1 criteria and compared to the baseline CT scan. In case of mesenteric mass at baseline, growth is defined as increase of ≥ 20% and ≥ 5 mm on the

short axis of the dominant mesenteric mass. In case of no mesenteric mass at baseline, growth is defined as development of a mesenteric node of ≥ 10 mm on the short axis.
